# Enhancing a *Sphaerobacter thermophilus* ω-transaminase for kinetic resolution of β- and γ-amino acids

**DOI:** 10.1186/s13568-023-01623-x

**Published:** 2023-10-21

**Authors:** Uwe Wegner, Falko Matthes, Nicolaus von Wirén, Ina Lemke, Rüdiger Bode, H.-Matthias Vorbrodt, Marion Rauter, Gotthard Kunze

**Affiliations:** 1 Leibniz Institute of Plant Genetics and Crop Plant Research (IPK), Corrensstr. 3, 06466 Seeland, OT Gatersleben, Germany; 2 Orgentis Chemicals GmbH, Bahnhofstr. 3-5, 06466 Seeland, OT Gatersleben, Germany; 3 Institute of Microbiology, University of Greifswald, Jahnstr. 15, 17487 Greifswald, Germany

**Keywords:** ω-Transaminase, *Sphaerobacter thermophilus*, Optimization, ß- and γ-amino acids, Kinetic resolution

## Abstract

*Sphaerobacter thermophilus* synthesizes an ω-transaminase (ω-TA) that allows the production of enantiomerically pure β-amino acids. To obtain ω-TA variants with a higher activity and more favorable properties for industrial use, we modified critical amino acid residues either in the catalytic center or in a previously proposed signature motif critical for aromatic β-amino acid ω-TAs. Seventeen different variants of this enzyme were generated and their activity was examined with four β-amino acids and one γ-amino acid, and compared with the wildtype’s activity. Among all variants, seven showed up to ninefold higher activity with at least one of the tested substrates. For most of these seven variants, the temperature optimum was even lower as in the wild type enzyme, with keeping a high temperature stability, making them more valuable for industrial purposes. Our results indicate that for the production of enantiomerically pure β-amino acids replacement of critical amino acid residues in the proposed signature motif of ω-TAs is a more effective strategy than modifying their catalytic center. Another finding was, that the proposed motif is not only suitable for aromatic amino acid ω-TAs, because some of the variants have a higher activity with β-alanine or β-leucine than with aromatic β-amino acids.

**Graphical Abstract:**

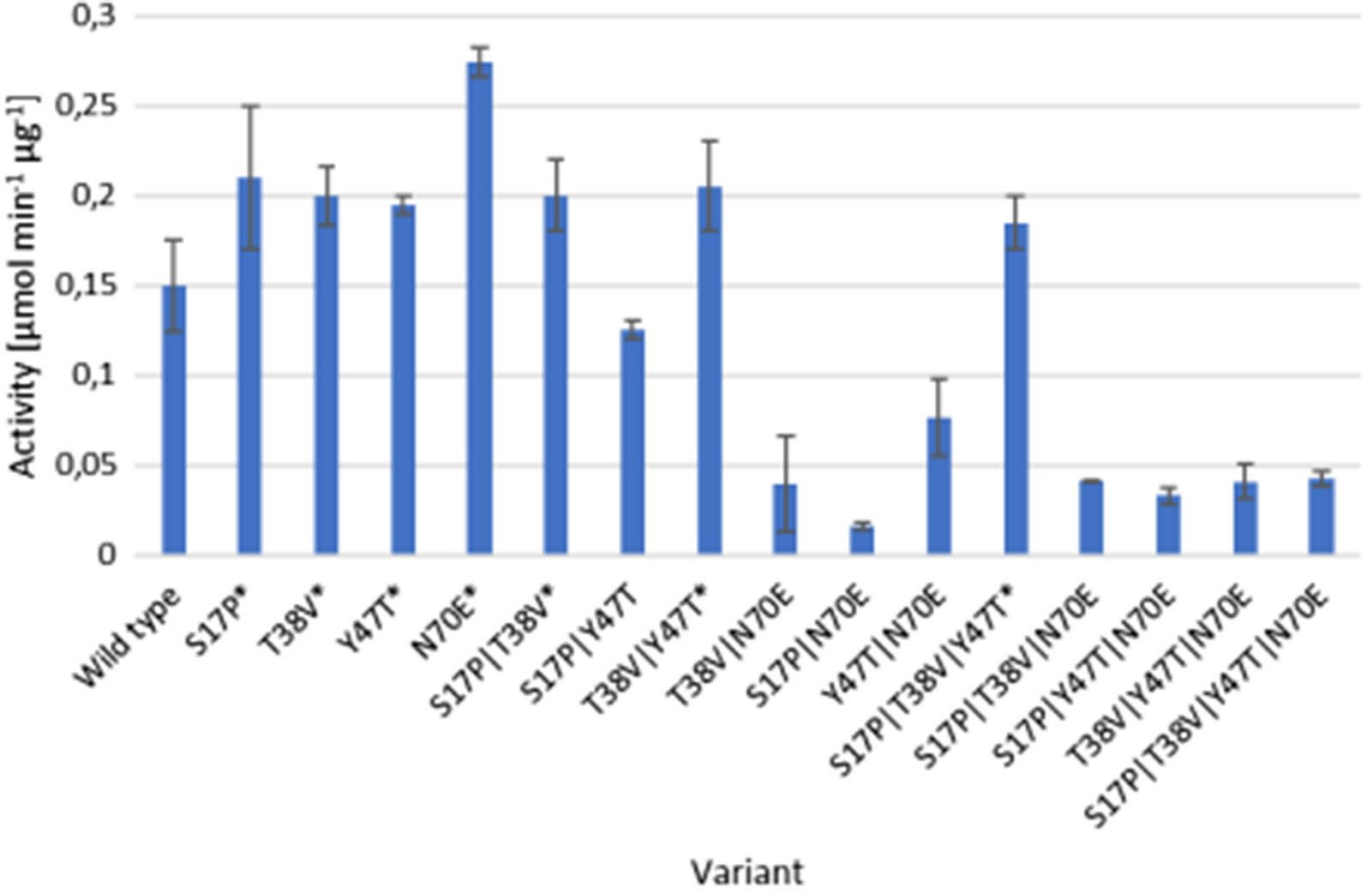

**Supplementary Information:**

The online version contains supplementary material available at 10.1186/s13568-023-01623-x.

## Key Points

- An ω-transaminase was modified to enhance its velocity in kinetic resolution.
- 17 variants were created and their properties were compared with the wildtype.
- 7 of them have a higher reaction speed with different β-amino acids as the wildtype.

**Supplementary Information:**

The online version contains supplementary material available at 10.1186/s13568-023-01623-x.

## Introduction

In all organisms, amino acids play a central role as building blocks of proteins and occur as intermediates in nitrogen (N) metabolism. Next to the proteinogenic α-amino acids, there also exist β- and γ-amino acids in which the amino group is shifted from the α-carbon to the adjacent β- or γ-carbon. They are not used in ribosomal protein synthesis, but take over specialized functions as secondary metabolites. For instance, certain rice cultivars secrete β-tyrosine in their root exudates to inhibit the growth of competing dicots or microbes (Yan et al. 2015). Other β- and γ-amino acids are components of secondary metabolites, such as β-alanine that is the most abundant β-amino acid and part of Coenzyme A (Juaristi and Soloshonok 2005). Others are part of complex secondary metabolites, like paclitaxel and lidamycin, which are used as therapeutics against several kinds of cancer (Wani et al. 1971; Hu et al. 1988) or to treat neurological dysfunctions (Crawford et al. 1987; Goa and Sorkin 1993; Field et al. 2001; Jääskeläinen 2005; Hamandi and Sander 2006). Another function of β- and γ-amino acids is found in the synthesis of peptidomimetics (Cabrele et al. 2014; Fujino et al. 2016), which are peptides consisting of β- and γ-amino acids whose secondary structures are similar to those of peptides made of their α-amino acid analogues (Iverson 1997; Koert 1997; Seebach et al. 1996; Seebach and Matthews 1997; Szefczyk 2021). The fact that these peptidomimetics are often less degradable by proteolytic enzymes (Hintermann and Seebach 1997; Frackenpohl et al. 2001; Gopi et al. 2003; Hook et al. 2004), increases their half-life as therapeutics.

The chemical synthesis of β- and γ-amino acids is mainly hampered by the poor stereo selectivity, which requires a cost-intensive separation of enantiomers (Weiner et al. 2010). On the other hand, a direct enantiopure synthesis of β- and γ-amino acids depends on expensive, and sometimes toxic, catalyzing agents and chiral-specific auxiliary materials (Liu and Sibi 2002). A promising alternative to chemical pathways is the enzymatic synthesis by transaminases (TAs).

**Fig. 1:**
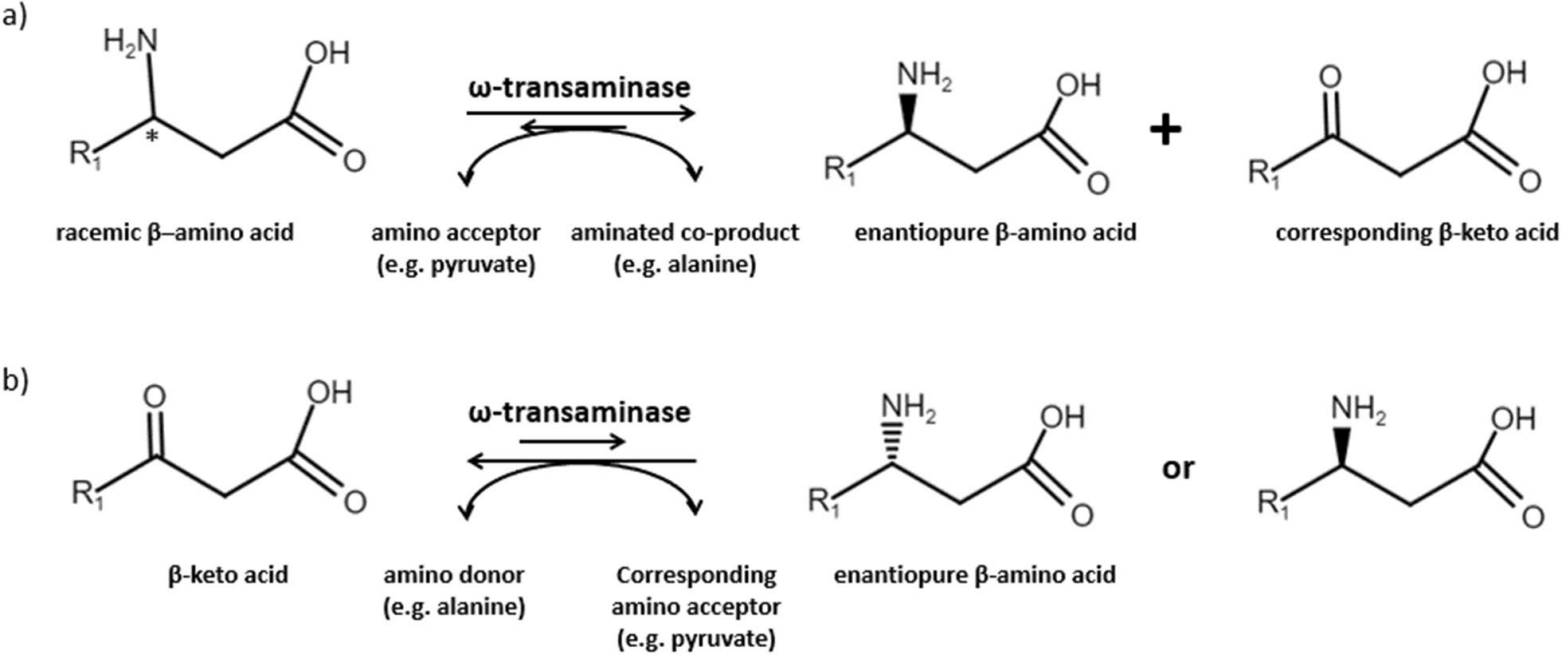
Reaction mechanisms for amino transferases converting β-amino acids. **a** Kinetic resolution, starting from a racemic β-amino acid to de-aminate one enantiomer and to transfer the amino group to an amino acceptor, in this case pyruvate. **b** The asymmetric synthesis starts with a prochiral β-keto acid, which is aminated and leads to an enantiopure amino acid. The asymmetric synthesis leads to the other enantiomer, concluding the kinetic resolution

In general, two reaction modes are suitable for the use of TAs: the kinetic resolution and the asymmetric synthesis (Shon et al. 2014). During the kinetic resolution, a racemic mixture is purified by stereo-selective degradation of one of the enantiomers. While making an expensive purification or the use of chiral-specific agents dispensable the drawback is a yield reduction by 50%. The asymmetric synthesis starts from a prochiral compound, e.g. a β-keto acid. This approach can lead to higher yields, but substrates as β-keto acids are not stable. In aqueous solutions, they tend to lose their carboxylic group (Bach and Canepa 1996; Xue et al. 2018). A valid alternative is the use of β-keto esters. By hydrolyzing the ester immediately before the transamination, it has been possible to produce β-phenylalanine from ethyl benzoylacetate (Buß et al. 2018). Another problem for the asymmetric syntheses is the unfavorable reaction equilibrium, laying on the side of the reactants (Fuchs et al. 2015). In principle, both enantiomers can be produced by using an ω-TA with high enantioselectivity. One enantiomer is produced by the kinetic resolution and the other by using the same enzyme for the asymmetric synthesis. Figure 1 presents the two reaction modes exemplary for the production of a β-amino acid, which work in the same way for γ-amino acids, aldehydes or ketones.

**Fig. 2:**
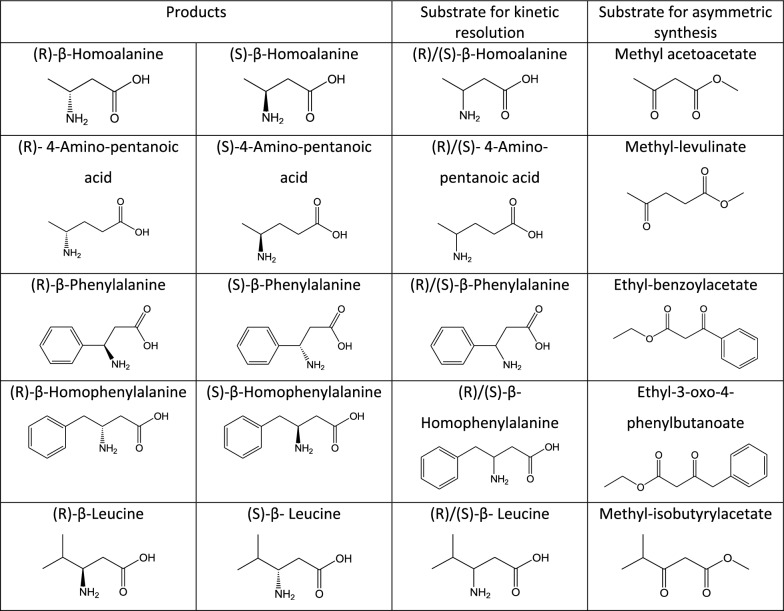
Compounds used for enzymatic catalysis by StoTA (*Sphaerobacter thermophilus* ω-transaminase). Given are substrates for the kinetic resolution and the asymmetric synthesis, as well as the corresponding products

Recently, a thermostable ω-TA from *Sphaerobacter thermophilus* has been described*,* which is able to use a broad range of substrates (Mathew et al. 2016) (RCSB (Berman et al. 2000) accession: 6K8H; NCBI accession: WP_012871332.1), hereinafter called StoTA (*Sphaerobacter* *thermophilus* ω-transaminase). A preliminary enzyme activity assay confirmed that this enzyme is able to turn over a wide range of different substrates (Fig. 2). Among these were several aromatic, a branched-chain and an aliphatic β-amino acid as well as an aliphatic γ-amino acid. Though StoTA was able to transform all substrates, the reaction velocity was rather low (Table 1). We subsequently tried to improve the enzyme’s turnover rate by a number of genetic modifications to increase its application for industrial use. For this purpose, we employed two approaches to generate StoTA variants with improved enzymatic properties, either by substituting critical residues in its catalytic center or by modifying critical residues in a motif that has been proposed to be critical for aromatic ω-TAs. In total, we generated 17 variants of StoTA and determined their specific activity with five substrates, as well as their pH, temperature optima and temperature stability. The aim of this study was to obtain StoTA variants with a higher activity and more favorable properties for industrial use.

**Table 1:** Comparison of the activities of different StoTA variants

Variant	β-HF (U mg^−1^)	β-HA (U mg^−1^)	4-AP (U mg^−1^)	β-L (U mg^−1^)	β-F (U mg^−1^)
WT	0.600 ± 0.029	0.300 ± 0.010	0.125 ± 0.015	0.130 ± 0.020	0.186 ± 0.060
T38V	0.610 ± 0.053	0.251 ± 0.015	0.106 ± 0.004	0.191 ± 0.000	0.251 ± 0.017
S17P	0.591 ± 0.035	0.388 ± 0.017	0.176 ± 0.008	0.093 ± 0.003	0.234 ± 0.003
N70E	0.802 ± 0.018	0.317 ± 0.005	0.114 ± 0.003	0.379 ± 0.001	0.362 ± 0.007
Y47T	3.007 ± 0.017	1.981 ± 0.043	0.871 ± 0.168	0.736 ± 0.125	1.141 ± 0.169
S17P|T38V	3.211 ± 0.009	2.226 ± 0.208	1.146 ± 0.037	1.056 ± 0.008	1.390 ± 0.021
T38V|Y47T	2.305 ± 0.026	1.591 ± 0.112	0.737 ± 0.014	1.369 ± 0.080	1.227 ± 0.025
S17P|T38V|Y47T	0.869 ± 0.016	0.429 ± 0.012	0.127 ± 0.003	0.386 ± 0.025	0.342 ± 0.033

Only modifications showing a higher activity with at least one substrate compared to the wild type (WT) are mentioned here. The specific activities with different amino donors are given with SD, N = 3

*β-HF* β-homo-phenylalanine, *β-HA* β-homo-alanine, *4-AP* 4-aminopentanoic acid, *β-L* β-leucine, *β-F* β-phenylalanine

## Material and methods

To illustrate the protein structure of the StoTA wild type and its variants for the motif adaption, ChimeraX was employed (Goddard et al. 2018). The crystal structure of StoTA is available at the protein data bank (Berman et al. 2000), accession 6K8H (Kwon et al. 2019). Conformational changes in the active center as depicted in Additional file 1: Figure S1 were illustrated by employing Mol* (Sehnal et al. 2021), using the same structure, 6K8H.

**Fig. 3:**
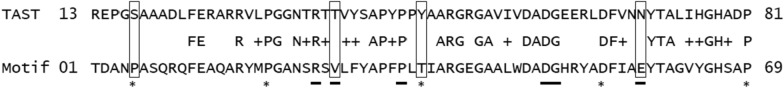
Blasted sequences of StoTA and motif (Crismaru et al. 2013). Identical and positive (+) residues are given in the middle line. Residues, which are underlined, are supposed to be important for ω-TA activity. Asterisks (*) mark residues, bordering secondary structures. Boxes indicate amino acids, which were exchanged, to match the motif

The coding sequence of StoTA was purchased as gene fragments from Eurofins (Eurofins, Luxemburg), together with all primers used in this work. Prior to ordering, the sequence was adapted to *E. coli*’s codon usage, by an in-house script provided by Eurofins (https://eurofinsgenomics.com/en/orderpages/genesynthesis/ordergenes/). A blast search (Altschul et al. 1997, 2005) of the putative motif (Crismaru et al. 2013) against the StoTA sequence (Fig. 3) was performed to select the residues to be substituted.

Primers were designed with an 18-base overlap (see Additional file 1: Table S1), to allow an isothermal assembly (Stemmer et al. 1995; Gibson 2011) after PCR. Each primer contained a single triplet, which differed from the template. PCR reactions were carried in a thermo cycler (Eppendorf Epgradient S Mastercycler), with an initial denaturation step at 98 °C for 3 min, followed by 35 cycles, each starts with a 10 s step at 98 °C. This was followed by a 30 s annealing step. The annealing temperatures can be found in Additional file 1: Table S2. At the end of each cycle, an elongation step at 72 °C for 1.5 min was performed. After the last cycle a final elongation at 72 °C for 10 min was done. After this step the temperature was hold at 4 °C.

PCR amplicons were cleaned by agarose gel electrophoresis and used for isothermal assembly. For assembly NEBuilder® HiFi DNA Assembly Cloning Kit (New England Biolabs, Ipswich, MA USA) was used, following the manufacturers protocol. The assembled genes were brought into pET 21 b (+) vector (Novagen, Merck, Darmstadt, Germany), using *Xho*I and *Nde*I (Thermo Scientific, Bremen, Germany). PET 21 b (+) provides a His-tag, directly behind the *Xho*I cleavage site, which was needed for subsequent purification of the ω-TAs.

The following substitutions were created: Single mutants: S17P, T38V, Y47T, and N70E; double mutants: S17P|T38V, S17P|Y47T, S17P|N70E, T38V|Y47T, T38V|N70E, Y47T|N70E; triple mutants: S17P|T38V|Y47T, 17P|Y47T|N70E, S17P|T38V|N70E, T38V|Y47T|N70E; and the quadruple mutant S17P|T38V|Y47T|N70E.

After plasmid assembly, the individual StoTA constructs were amplified in *E. coli* XL1 blue (Stratagene CA, USA). Transformation and selection were performed according to manufacturer’s protocol. The plasmids were isolated and transferred into *E. coli* BL21(DE3) cells (Stratagene CA, USA). Positive transformants were identified in the same way as in XL1 blue. 50 ml of LB-Amp medium were inoculated to obtain an OD_600 nm_ of 0.075. The cultures were shaken (37 °C, 180 rpm) for 2 h. For each construct, two samples were made, one which was induced with 0.5 mM IPTG, another as negative control without IPTG. Thereafter, cultures were shaken at 37 °C and 180 rpm for another 2 h. After this step, cells were harvested and lysed in bugbuster® (Merck, Darmstadt, Germany) following the manufacturer’s protocol and centrifuged for 5 min at 4 °C and 16,000*g*. The supernatant containing the soluble protein fraction was separated from the pellet. The latter, containing insoluble protein fraction and cell debris was resuspended in 250 µl of TE-buffer pH 7.4. 20 µl of all fractions were used for one-dimensional SDS-PAGE (Laemmli 1970). After electrophoresis, Western blots were performed using a PVDF membrane (Towbin et al. 1979). To figure out which samples contained the modified variants of StoTA, membranes were stained by antibodies selective for the His-tags. The rest of the supernatant was used for the extraction of the StoTA variants via Ni–NTA (Abcam, Cambridge, UK), again following the manufacturer’s protocol. The protein concentration of the extracts was determined with Roti Nanoquant® (Carl Roth, Karlsruhe, Germany) following the manufacturers protocol for micro titer plates.

### Activity assay

Activity assays were performed to compare the activity of the wild type StoTA enzyme with the mutated variants. To measure enzyme activities 10 µl of purified enzyme solution was added to 90 µl of reaction medium containing 10 mM amino donor (all from TCI, Tokyo, Japan), 4 mM pyruvate (Boehringer-Mannheim, Mannheim, Germany), 0.1 mM PLP (Merck, Darmstadt, Germany) and 0.1 M potassium-phosphate buffer pH 8.0. After incubation for 30 min at 40 °C, 17.5 µl of the sample was diluted with 52.5 µl water and 70 µl of 1 mM 2,4-dinitrophenylhydrazine (1 mmol l^−1^, Fluka, Buchs, St. Gallen) dissolved in 1 M HCl (Carl Roth, Karlsruhe, Germany), followed by incubation for 20 min at room temperature. Following the addition of 70 µl of 4 M NaOH the absorption was measured at 570 nm (TECAN infinite® 200 plate reader) to quantify the remaining pyruvate. As amino donors β-homophenylalanine, β-phenylalanine, β-homoalanine, β-leucine and 4-aminopentanoic acid (all purchased from TCI, Tokyo, Japan) were used. Since pyruvate and the amino donor react in a 1:1 stoichiometry, the amount of deaminated amino donor was determined. The specific activity of StoTA was calculated from the amount of deaminated amino donor, referred to the reaction time and expressed in µmol * min^−1^ * µg^−1^.

Mutant enzymes with higher activity than that of the wild type were used for further tests to determine the optimal pH value and the temperature stability. The determination of pH optimum and temperature stability was similar to the activity assay. For the pH optimum, different buffers between pH 2 and pH 10 (Additional file 1: Table S3) were used instead of the standard buffer, while β-homophenylalanine was used as amino donor. To determine temperature stability, aliquots of the purified enzymes were stored for 60 min at 30–90 °C, in 10 °C steps before performing the activity assay. To determine the temperature optimum, the activity was measured for 30 min at 30–80 °C, again in 10 °C steps. Tests for pH and temperature optimum and temperature stability were performed with β-homophenylalanine as amino donor.

### In silico structure prediction

The structures of the mutant enzymes were predicted by using AlphaFold (Jumper et al. 2021) on a colab (Mirdita et al. 2021) notebook (https://colab.research.google.com/github/sokrypton/ColabFold/blob/v1.2.0/AlphaFold2.ipynb). For the wild type and each mutant, the number of calculated models was reduced to four, all other settings were used at default values. The ‘use template’ function was not used. For the illustrations (Additional file 1: Figure S2), the models with the best rank were used.

## Results

### In silico modelling

**Fig. 4:**
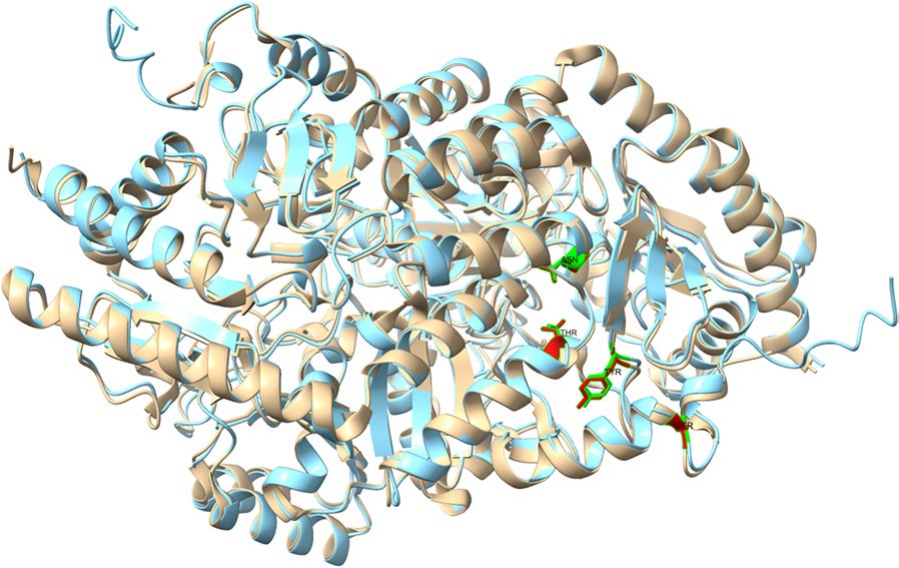
Alignment of the crystal structure and the in silico predicted structure of StoTA. Brown: crystal structure, blue: AlphaFold predicted structure. The four amino acids, which are exchanged are marked in green/red and labeled

Comparing the in silico modelled structure of the wild type enzyme with the crystal structure, we found both structures to be very similar (Fig. 4). This indicates the high reliability of the in silico modelling procedure which showed that all mutant enzyme had the same structure, no differences in folding could be observed (see Additional file 1).

### Modification of the active center of StoTA

The substitution of I283 and I284 in the catalytic domain by glutamine resulted in a decrease of activity by approximately 54% with β-homoalanine, a loss of approximately 49% with β- phenylalanine and an almost complete loss of activity with 4-aminopentanoic acid and β-leucine. The remaining activity against β-homophenylalanine was only 76.4% compared to the wild type enzyme. Exchanging T317 with valine led to a loss of activity. T317V was not active with β-homophenylalanine, β-homoalanine and β-leucine. With β- phenylalanine the remaining activity was only 26.3%, compared to the wild type. Only with 4-amino-pentanoic acid T317V showed a similar activity as the wild type. Consequently, the approach to modify the active center was not continued.

### Modification of the proposed signature sequence motif in StoTA

**Fig. 5:**
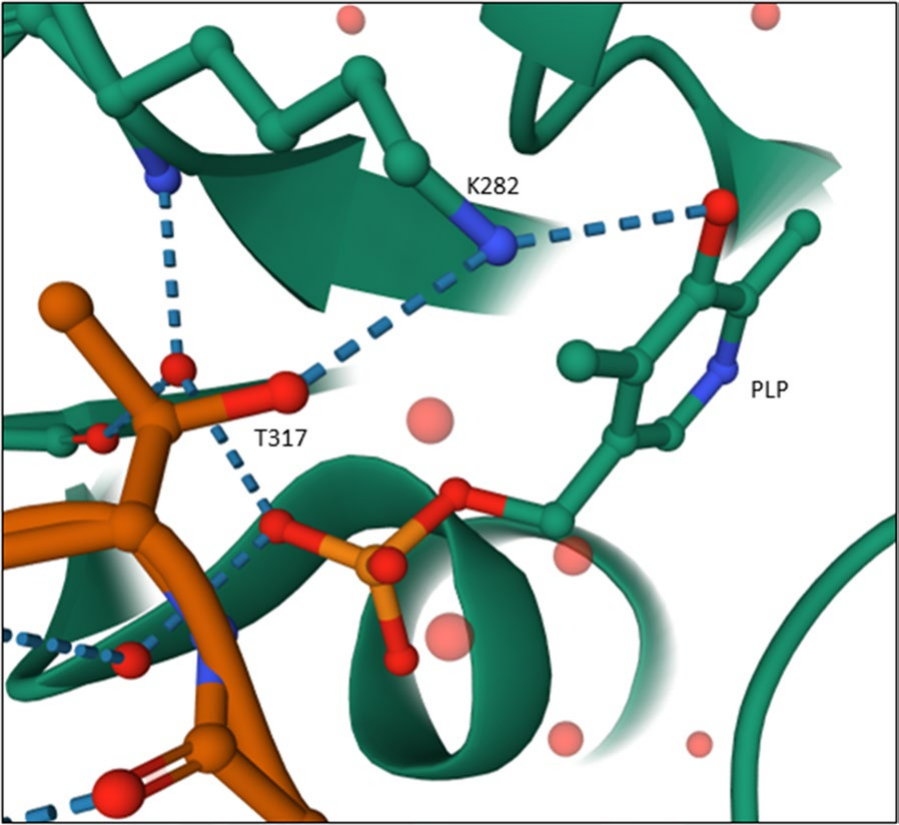
Results of the pre-test with crude extracts. Bars indicate the activity of 1 mg crude extract of each variant with β-homophenylalanine with SD. N = 3. The 7 variants with a higher activity than the wild type are marked with an asterisk (*)

The replacement of 4 amino acids was necessary to adapt StoTA to a proposed motif (Crismaru et al. 2013) (Fig. 3). At all 15 different variants of StoTA were generated, the variants were: S17P, T38V, Y47T, N70E, S27P|T38V, S17P|Y47T, S17P|N70E, T38V|Y47T, T38V|N70E, Y47T|N70E, S17P|T38V|Y47T, S17P|T38V|N70E, S17P|Y47T|N70E, T38V|Y47T|N70E and S17P|T38V|Y47T|N70E. Pre-tests with crude extracts from all variants were performed with β-homo-phenylalanine. Seven variants (marked with an asterisk in Fig. 5) which had a higher activity were used for further characterization. In the ongoing experiments purified enzymes were used. The specific activities, as well as several biochemical properties are given in Tables 1 and 2.

**Table 2:** Temperature stability is the temperature which does not inactivate the enzyme after 60 min of incubation, or 24 h, respectively

Variant	pH optimum	Temperature optimum (°C)	Temperature stability 1 h (°C)	Temperature stability 24 h (°C)
WT	8.0	60	60	40
T38V	7.0	60	50	50
S17P	7.0	40	50	40
N70E	8.0	40	60	40
Y47T	8.0	48	50	40
S17P|T38V	8.0	40	50	40
T38V|Y47T	8.0	46	50	40
S17P|T38V|Y47T	8.0	40	50	40

Only modifications which show a higher activity with at least one substrate compared to the wild type (WT) are listed here

### Temperature stability, temperature optimum and pH-optimum

The modified versions of StoTA retained their activities also at higher temperatures. Like the wild type enzyme, the N70E variant was stable for 60 min at up to 60 °C, while the remaining mutants were stable for 60 min at up to 50 °C. In most mutants, the optimum temperature was lower than for the wild type, i.e. 60 °C. For the mutants S17P, N70E, S17P|T38V and S17P|T38V|Y47T this optimum was only 40 °C, for mutant T38V|Y47T 46 °C and for mutant Y47T 48 °C. Only T38V had a similar temperature optimum as the wild type.

Amino acid substitutions in StoTA had no noteworthy effect on the pH optimum. Both wild type enzyme and the variants Y47T, N70E, S17P|T38V, T38V|Y47T and S17P|T38V|Y47T worked best at a pH value of 8. Only T38V and S17P showed a shift of their pH optimum to pH 7.

## Discussion

### Modification of the active center of StoTA

**Fig. 6:**
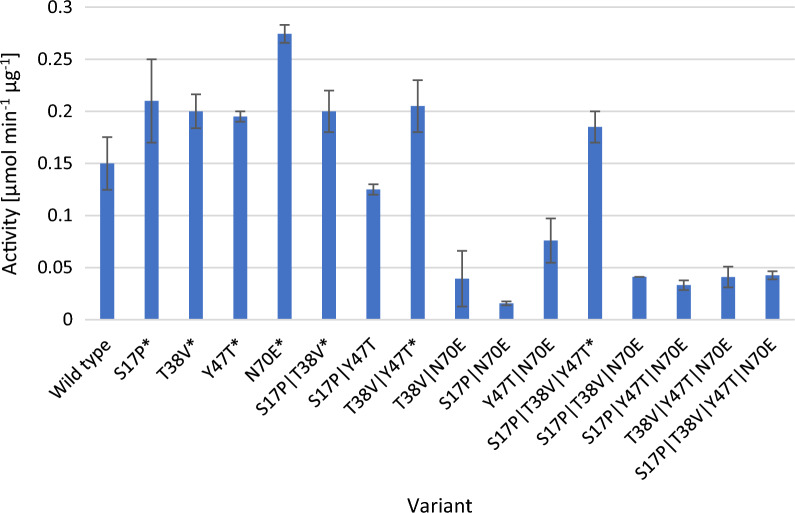
Interaction between T317 and K282 in StoTA, data for the interactions are stored in the model 6K8H. Dotted blue lines indicate hydrogen bonds between residues and the co-factor PLP. The side chain of T317 forms a hydrogen bond to the side chain of K282, while K282 forms another hydrogen bond to the carbonyl group of PLP. Green: chain A, orange: chain B. Red dots are water molecules

In general, StoTA was modified in two ways. We first focused on the active center, in which a lysine residue at position 282 (K282) is part of the catalytic domain that performs a nucleophilic attack on the carbonyl group of PLP to form a Schiff’s base (Crismaru et al. 2013). Under physiological conditions, the amino group of the sidechain of lysine is protonated, which makes it a weaker nucleophile. To prevent this protonation, the environment of K282 was made more alkaline. For this purpose, two amino acids in the vicinity of the catalytic lysine (I283 and I284) were replaced by glutamine. The second modification concerned the replacement of an interacting threonine residue (T317), which by forming a hydrogen bond to K282, might pull the latter away from PLP (Fig. 6). To confirm this, T317 was substituted by valine and the resulting conformational changes predicted by structural modelling (Additional file 1: Figure S2).

In general, StoTA variants with substitutions in the active center showed a similar or lower activity as the wild type enzyme (Fig. 5). T317V was not active at all with β-homophenylalanine, β-homoalanine and β-leucine. Compared to the unmodified enzyme, the activity with β-phenylalanine also decreased to 26.3%. Only with 4-amino-pentanoic acid T317V showed a similar activity as the wild type. This confirms that residue T317 should be part of the suggested motif (Crismaru et al. 2013), or even the active center. T317 of chain A is proposed to interact with K282 of chain B, and vice versa (Fig. 6). Assuming that T317 is important for the dimerization of the enzyme, a loss of T317 will lead to a more unstable and less active protein complex. Amongst at least five other residues, T317 is also involved in PLP binding (Kwon et al. 2019). This could be another reason for the loss of activity, if the missing hydrogen bond between T317 and the phosphate group of PLP leads to an incorrect binding of the co-factor. Another possibility is that T317 is not drawing K282 away from the active center but is bending or stabilizing K282 in a position where it can bind the keto group of PLP or another substrate.

### Modification of the proposed signature sequence motif in StoTA

In the second approach, StoTA was modified to match the proposed signature sequence motif that allows aminotransferases converting aromatic β-amino acids (Crismaru et al. 2013). A blast search (Altschul et al. 1997) of the putative motif sequence against the StoTA sequence revealed that only four substitutions are required to fit StoTA to the proposed motif (Fig. 3). These were S17P, T38V, Y47T and N70E. We generated fifteen different variants for this type of modification. Four of those carried a single amino acid substitution, six carried two substitutions, four carried three substitutions, and one variant carried all four substitutions. Some of the modifications of StoTA led to an increased activity, emphasizing the importance of some of the amino acids in the proposed motif. Several mutants, which were made to adapt StoTA to the motif, had comparable activities to the wild type, others showed different activity levels.

The double substitution mutant T38V|N70E led to a loss of activity, showing approximately 75% activity with 4-aminopentanoic acid and β-phenylalanine and only 35% with β-leucine, while β-homophenylalanine and β-homoalanine were deaminated at almost the same velocity as with the wild type. In contrast, the double mutant, S17P|T38V showed a strong increase in activity. Depending on the amino donor, S17P|T38V displayed a 5.4 to 9.2-fold higher activity compared to the wild type. Also, T38V|Y47T was 3.8–10.5 times more active than the wild type. A strongly enhanced activity with all tested substrates was also found in the single mutant Y47T. Depending on the amino donor, the specific activity increased by 5.0–7.0 times compared to the wild type. This might be due to the fact that threonine has a less bulky side chain than tyrosine making it easier for a substrate molecule to enter the active center.

Overall, the double mutant S17P|T38V had the highest activity of all StoTA variants with all substrates, except for β-leucine. With β-leucine the double mutant T38V|Y47T fared best with a 10.5 times enhanced activity compared to the wild type. Interestingly, as single replacement T38V had a lower activity than the wild type for all substrates except β-leucine, while S17P had lower or similar activities with all substrates. Together, however, these substitutions exerted a strong influence on the enzyme activity. This is somewhat surprising considering that T38 seems too far away from either S17 or Y47 to provoke an interaction. The increase of activity must have other reasons, a difference in protein folding can be excluded based on in silico folding.

**Fig. 7:**
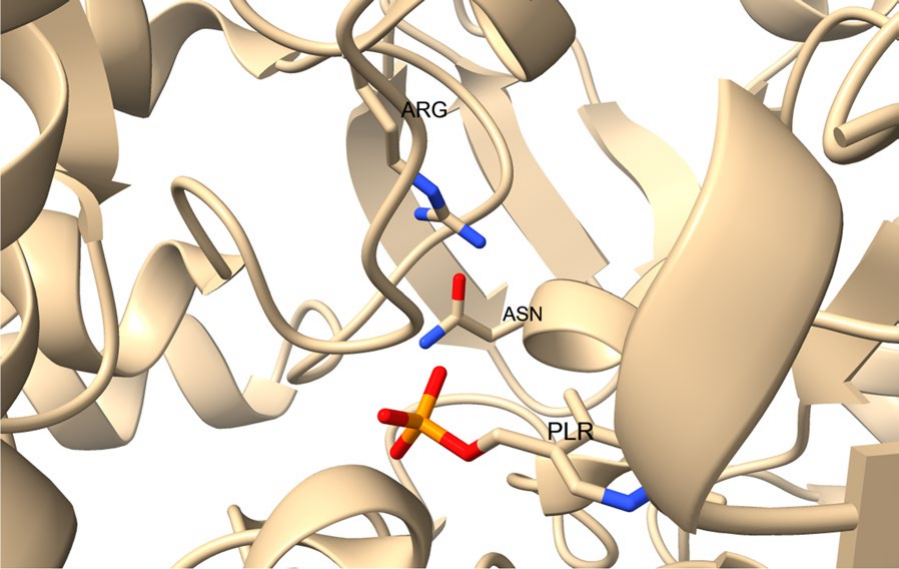
Close up to the active center of 6K8H. residues of N70 and R36 are shown and named

Mutant StoTA in which N70 was replaced by glutamate had a 9.1% loss of activity with 4-aminopentanoic acid thus revealing the importance of asparagine at position 70 for transforming γ-amino acids. Regarding β-amino acids, a different influence was observed. All β-amino acids were converted faster in comparison to the wild type (β-homoalanine + 29.3%, β-leucin + 191.5%, β-phenylalanine + 94.6% and β-homophenylalanine + 33.7%). The motif proposed by (Crismaru et al. 2013) was originally suggested only for aromatic amino acids. However, as observed here, fitting StoTA to the motif had a high impact on the activity with a branched chained amino acid and a minor impact on the activity with aromatic amino acids. This shows that the motif can be used for non-aromatic ω-Tas too. N70 is located close to the phosphate group of bound PLP, where it may interact with the phosphate of the bound PLP (Fig. 7). In the vicinity of N70, R36 might be the residue, which binds the carboxyl group of the substrate to hold it in the p-pocket. Exchanging N70 by glutamate obviously alters the interactions between this residue and PLP or R36. The altered interaction appears to be beneficial for some substrates, but disadvantageous for others.

Depending on the amino donor the quadruple mutant, S17P|T38V|Y47T|N70E, showed only 8.6–81.4% residual activity, suggesting that not all residues in the proposed motif are equally important. The highest gains of activity were achieved in the mutants Y47T, S17P|T38V and T38V|Y47T. Additionally, these mutants have a lower temperature optimum, ranging between 40 and 50 °C instead of 60 °C. This is relevant for industrial use, since a lower temperature optimum requires less energy thus reducing production costs.

Previously, the activity with β-phenylalanine was improved by site-directed mutagenesis of an ω-TA from *Caulobacter crescentus* (Hwang et al. 2008). Two variants of this enzyme, N285A and V227G showed a three and twofold activity increase, respectively, compared to the wild type. On the other hand, these variants had a four and twofold lower activity with β-homoalanine (Hwang et al. 2008). By introducing two mutations, (Cho et al. 2008) optimized the activity of an ω-TA from *Vibrio fluvialis* against aromatic and aliphatic amines. Depending on the amino donor, W57G had a 2- to 40-fold higher activity with aromatic amines and a 5- to 19-fold higher activity with aliphatic amines. In the second variant, W147G the activity with aromatic amines ranged between 0.8-fold to 14-fold relative to the wild type, while the activity with aliphatic amines ranged between 0.9- and 6-fold (Cho et al. 2008). These results are comparable to those of the best variants obtained in the present study. Although none of our variants reached a 40-fold activity gain as obtained for the *V. fluvialis* enzyme for one substrate (Cho et al. 2008), the best variants produced in this work showed a consistent activity rate across all substrates tested.

### Specific activity with different substrates

All variants of StoTA catalyzed the deamination of all tested substrates. The activities were measured with β-homophenylalanine, β-homoalanine, 4-aminopentanoic acid, β-leucine and β-phenylalanine. The highest specific activity with β-homophenylalanine was observed with the S17P|T38V variant in which 1 µg protein was able to convert 3.211 ± 0.009 µmol β-homo-phenylalanine in 1 min, which is defined as U µg^−1^. Y47T has a quite similar activity, 3.007 ± 0.017 U µg^−1^, and T38V|Y47T was able to deaminate 2.305 ± 0.026 µmol β-homo-phenylalanine per minute.

For β-homoalanine, S17P|T38V achieved the highest specific activity, namely 2.226 ± 0.208 U µg^−1^, followed by Y47T, 1.981 ± 0.043 U µg^−1^ and T38V|Y47T with a specific activity of 1.591 ± 0.112 U µg^−1^. With 4-aminopentanoic acid, S17P|T38V was the only variant with a specific activity above 1 U µg^−1^ (1.146 ± 0.037 U mg^−1^). With an activity of 1.369 ± 0.080 U µg^−1^, T38V|Y47T had the highest specific activity of all variants with β-leucine followed by S17P|T38V with a specific activity of 1.056 ± 0.008 U µg^−1^. Also, in case of β-phenylalanine, S17P|T38V had the highest specific activity, followed by T38V|Y47T.

Taken together, the present study shows that several biochemical properties of StoTA could be successfully improved by a rational design based on a previously proposed signature sequence motif for aminotransferases converting aromatic β-amino acids. Not all modifications lead to a higher enzyme activity, but 7 out of 17 variants (S17P, T38V, Y47T, N70E, S17P|T38V, T38V|Y47T and S17P|T38V|Y47T) showed a higher specific activity with at least one substrate. Among all variants, three modifications, Y47T, S17P|T38V and T38V|Y47T not only had, depending on the amino donor, an approx. 4–10.5 times higher activity than the wild type, the same mutants also had a lower optimal temperature of 40–50 °C. These properties make these three variants valuable candidates for industrial processes. Especially, S17P|T38V is a very promising modification, it has the highest specific activities with β-homophenylalanine, β-homoalanine, 4-aminopentanoic acid and β-phenylalanine of all tested variants and can be used to produce these amino acids. Additionally, S17P|T38V has a temperature optimum of only 40 °C, which is 20 °C lower than for the wild type, which makes this variant even more suitable for industrial processes. Only for the production of β-leucine, T38V|Y47T would be better because of the roughly 30% higher specific activity compared to S17P|T38V.

Besides the improvement of the catalytic activity, T317 could be identified as an essential residue. Without this residue, the enzyme loses most of its activity. The assumption that T317 is important for PLP binding is strengthened by the fact that PLP is not added to the model if the T317V variant is folded in silico by swiss-model (https://swissmodel.expasy.org/interactive) (Waterhouse et al. 2018), even if the unmodified structure, which includes PLP, is used as template.

### Supplementary Information

**Additional file 1: Figure S1.** Structure of the active center of StoTA. Wildtype (left) compared to the mutants T317V (upper right) and I238Q|I248Q (lower right). **Figure S2.** View at the active centers of StoTA. StoTA is a homodimer. Yellow: chain a, grey: chain b, orange: PLP bound to K282. The residues, which will be exchanged are colored in blue. The box marks the cutout which is presented in the following illustrations. **Table S1.** Used primers for the adaption to the motif. **Table S2.** Annealing temperatures for all primer pairs. **Table S3.** Used buffers for the determination of pH optima. All buffers had a concentration of 1 M.

## Data Availability

The data for Fig. 6, Tables 1, 2 and the AlphaFold predictions can be found at the e!DAL repository (https://edal-pgp.ipk-gatersleben.de/) (Arendt et al. 2014), under the DOI http://dx.doi.org/10.5447/ipk/2023/12. The crystal structure as well as the sequence of the wild type enzyme is available at RCSB.org under the accession number 6K8H. The sequence of the codon optimized wild type gene can be found at the e!DAL repository (preliminary address: https://doi.ipk-gatersleben.de/DOI/746cc46e-1641-47a7-b225-c14d107238db/8fabfe05-74fd-436a-9760-77db1ccccfe9/2/1847940088).
