# Supplementary material for: Enhancing a Sphaerobacter thermophilus ω-transaminase for kinetic resolution of β- and γ-amino acids
Source: AMB Express. 2023 Oct 21;13:117. doi: 10.1186/s13568-023-01623-x (PMC10589169; doi:10.1186/s13568-023-01623-x)
Supplement: Supplementary file 1 — Additional file 1: Figure S1. Structure of the active center of StoTA. Wildtype (left) compared to the mutants T317V (upper right) and I238Q|I248Q (lower right). Figure S2. View at the active centers of StoTA. StoTA is a homodimer. Yellow: chain a, grey: chain b, orange: PLP bound to K282. The residues, which will be exchanged are colored in blue. The box marks the cutout which is presented in the following illustrations. Table S1. Used primers for the adaption to the motif. Table S2. Annealing temperatures for all primer pairs. Table S3. Used buffers for the determination of pH optima. All buffers had a concentration of 1 M. [file 13568_2023_1623_MOESM1_ESM.pdf]

Applied Microbiology and Biotechnology

Enhancing a *Sphaerobacter thermophilus* ω-transaminase for kinetic resolution of β- and γ-amino acids

Uwe Wegner[1], Falko Matthes[1], Nicolaus von Wirén[1], Ina Lemke[1], Rüdiger Bode[3], H.-Matthias Vorbrodt[2], Marion Rauter[2], Gotthard Kunze[1]*

1. Leibniz Institute of Plant Genetics and Crop Plant Research (IPK), Corrensstr. 3, D-06466 Stadt Seeland OT Gatersleben, Germany.
2. Orgentis Chemicals GmbH, Bahnhofstr. 3-5, D-06466 Stadt Seeland OT Gatersleben, Germany
3. Institute of Microbiology, University of Greifswald, Jahnstr. 15, D-17487 Greifswald, D-17487 Greifswald, Germany.

* Corresponding author: Gotthard Kunze, Leibniz Institute of Plant Genetics and Crop Plant Research (IPK), Corrensstr. 3, D-06466 Stadt Seeland OT Gatersleben, Germany Tel. (+49) 39482-5401; e-mail: kunzeg@ipk-gatersleben.de

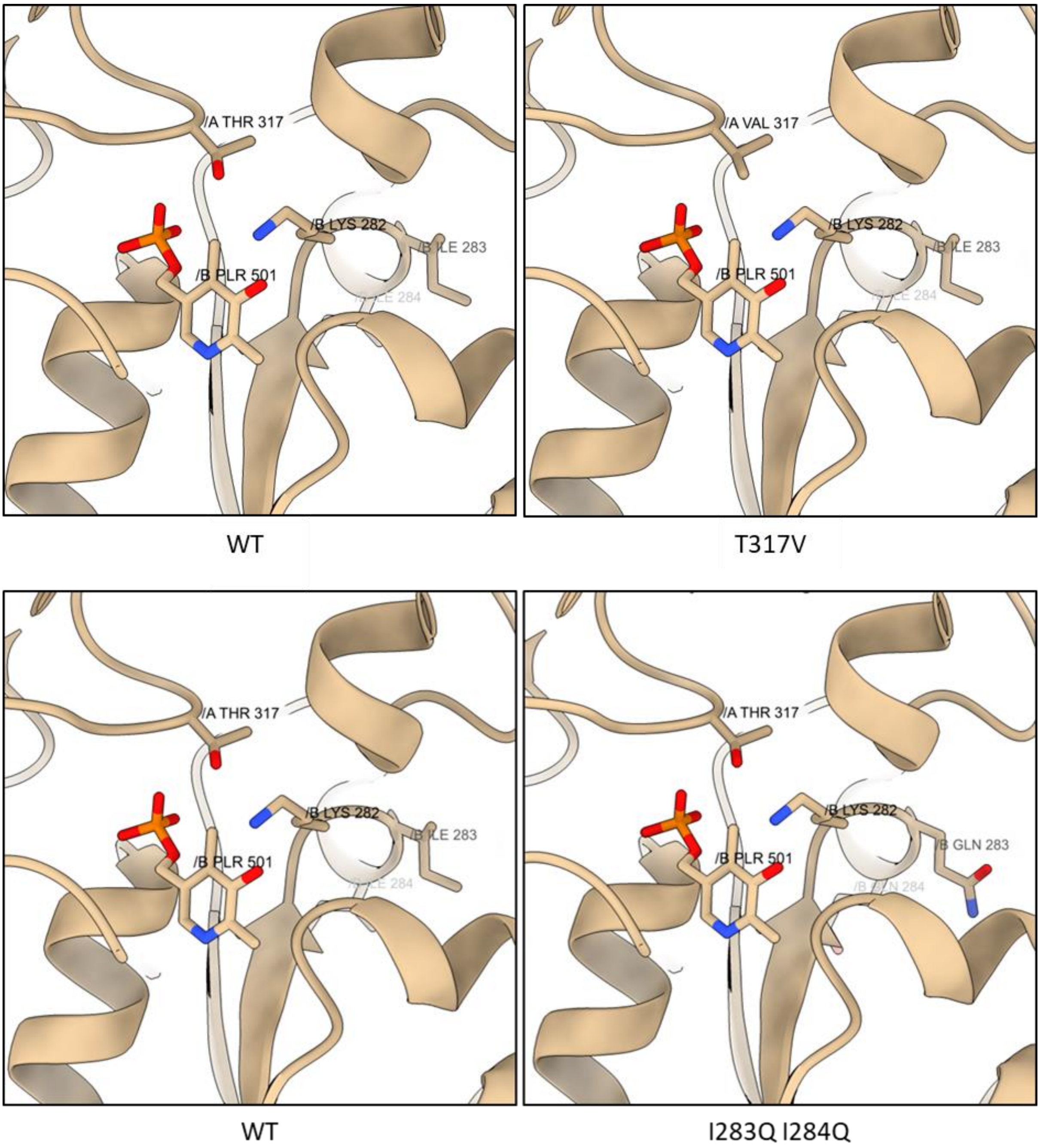

Figure S1: Structure of the active center of StoTA. Wildtype (left) compared to the mutants T317V (upper right) and I238Q|I248Q (lower right).

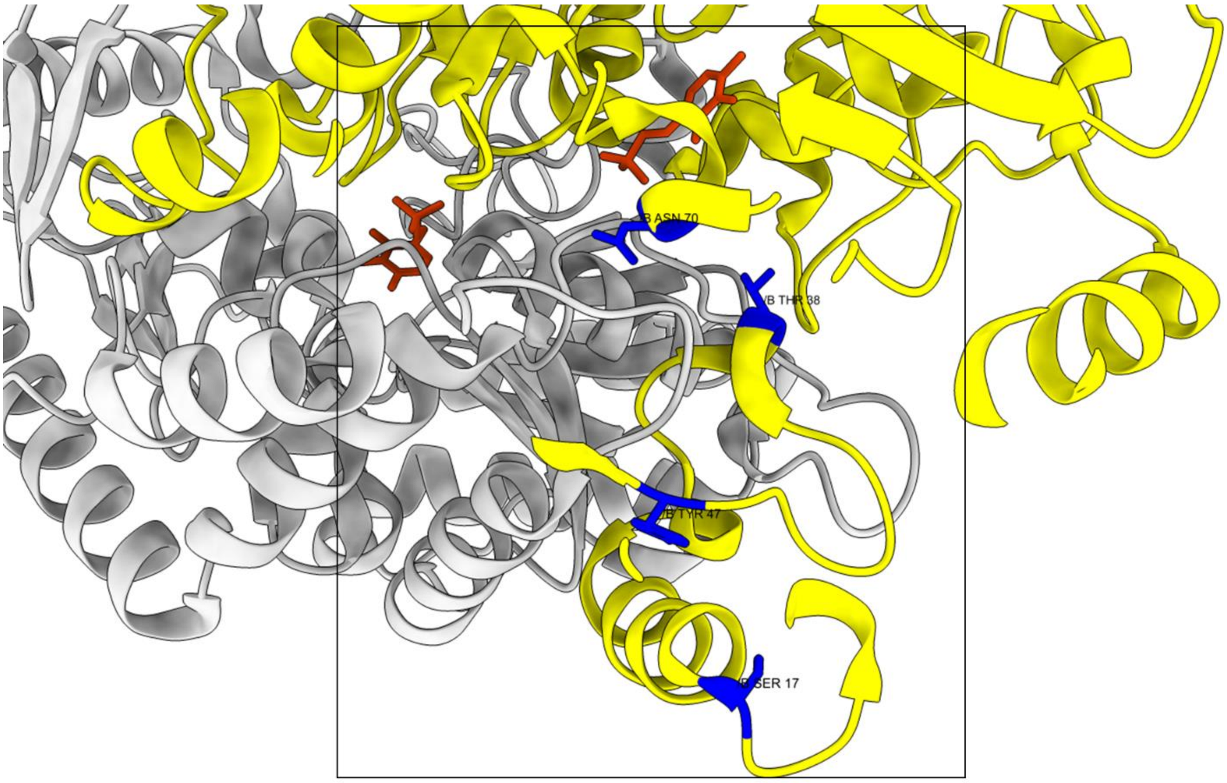

Figure S2: View at the active centers ofStoTA.StoTA is ahomodimer. Yellow: chain a, grey: chain b, orange: PLP bound to K282. The residues, which will be exchanged are colored in blue. The box marks the cutout which is presented in the following illustrations.

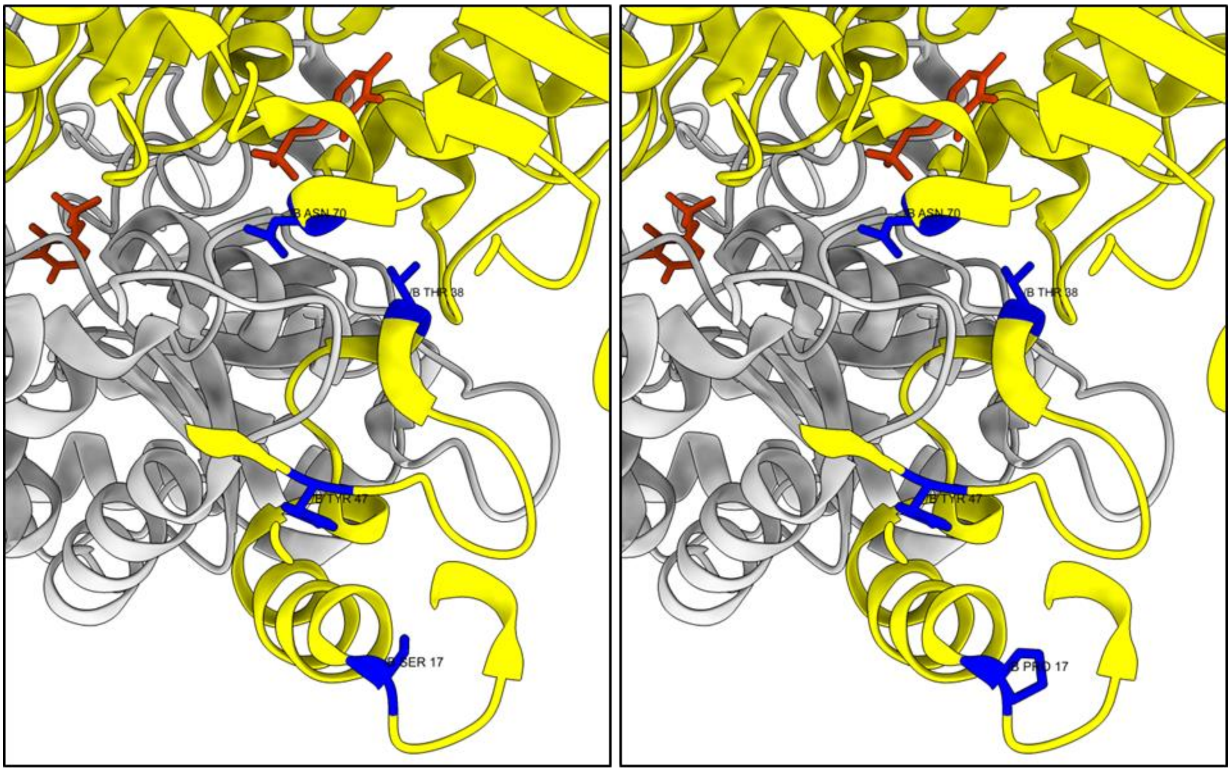

WT S17P

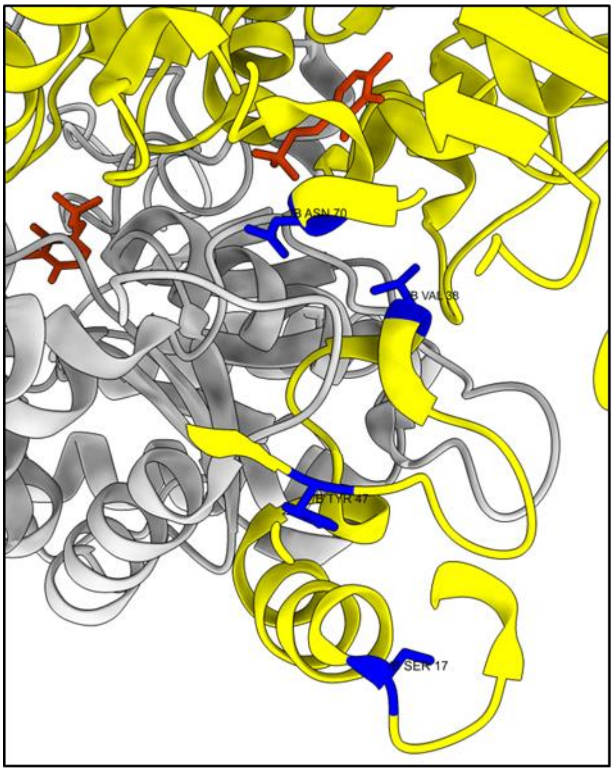

T38V

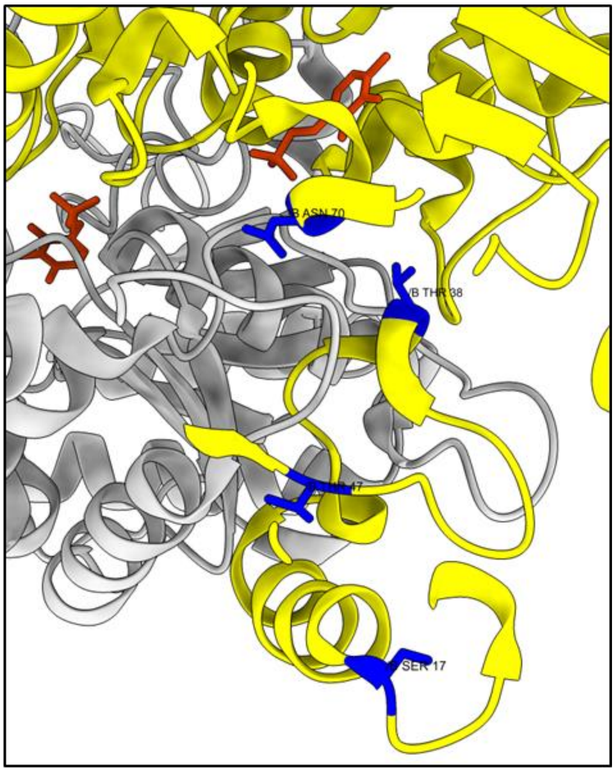

Y47T

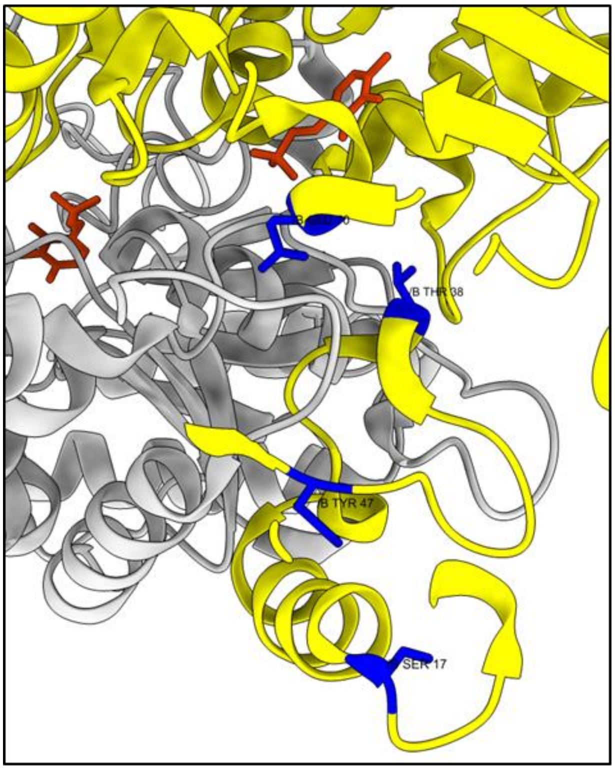

N70E

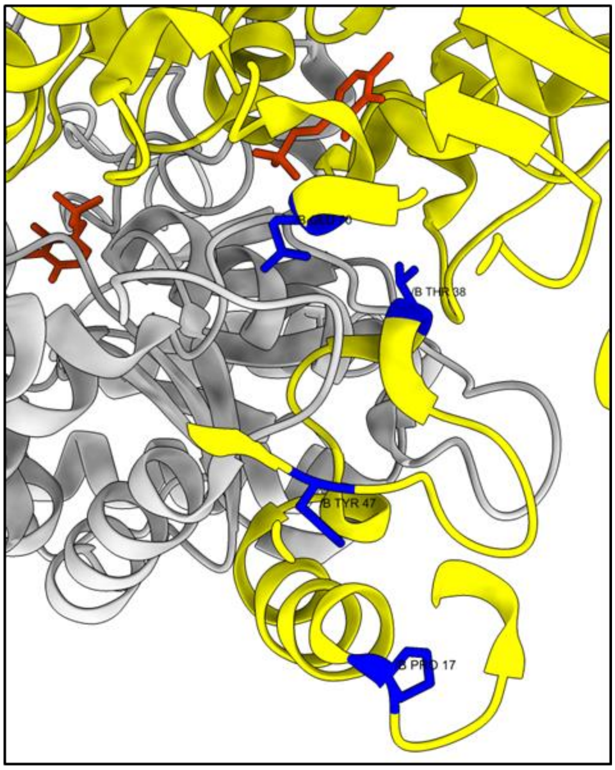

S17P N70E

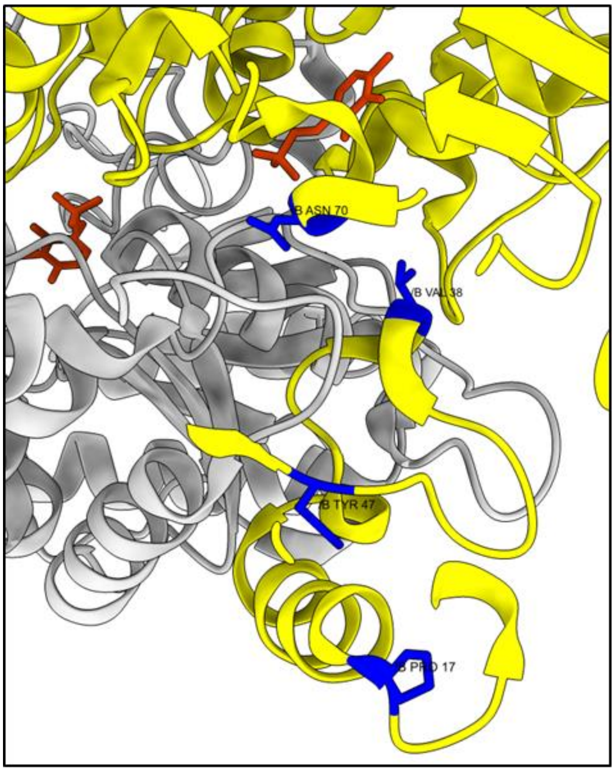

S17P T38V

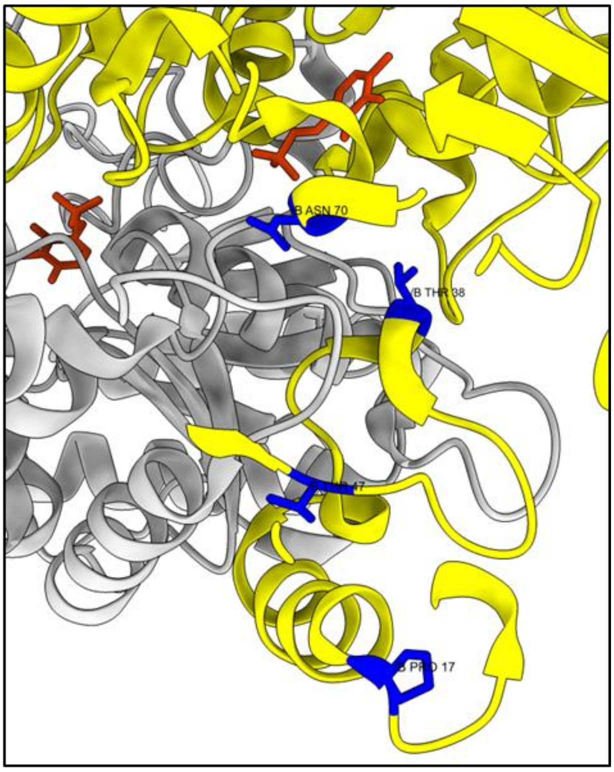

S17P Y47T

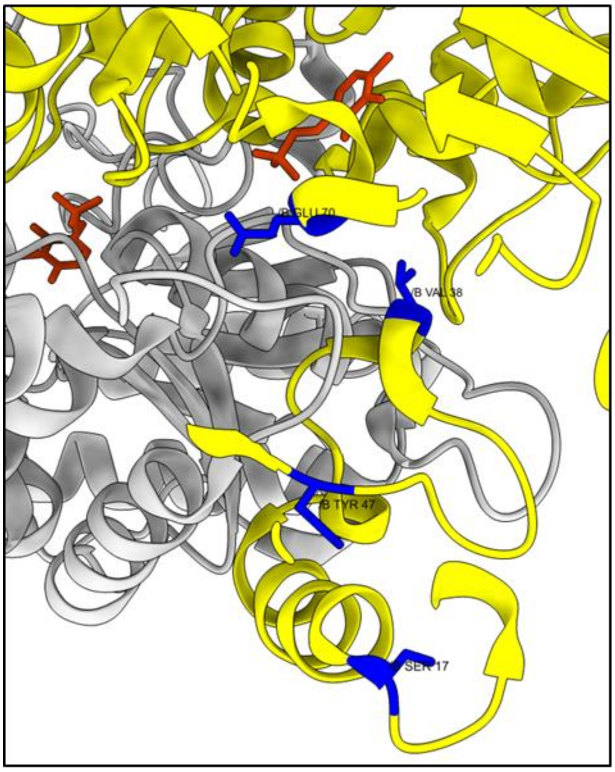

T38V N70E

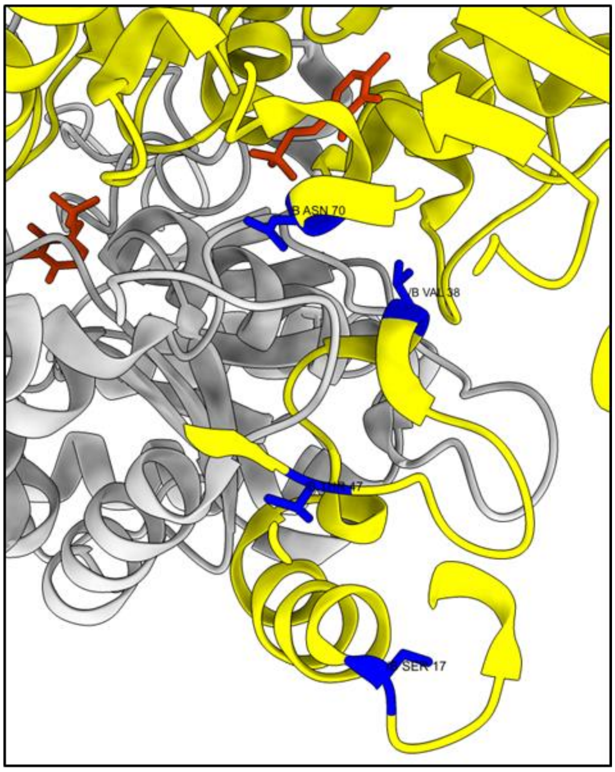

T38V Y47T

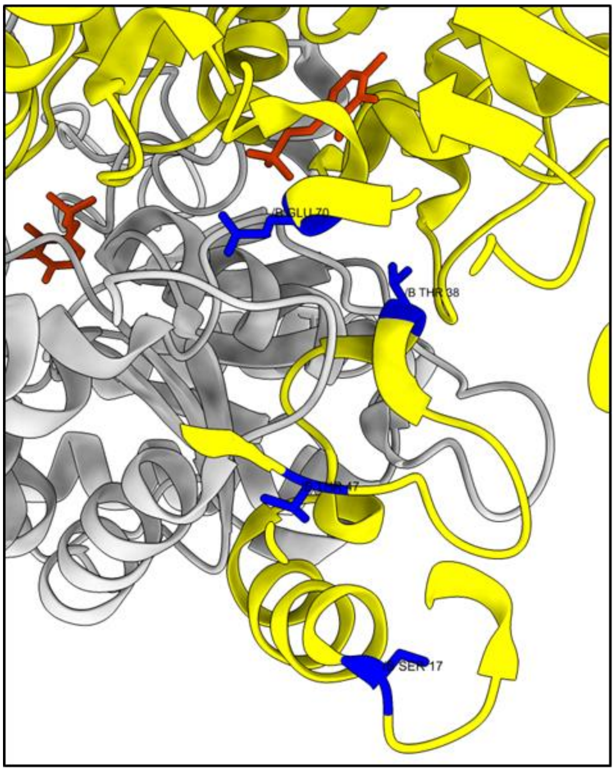

Y47T N70E

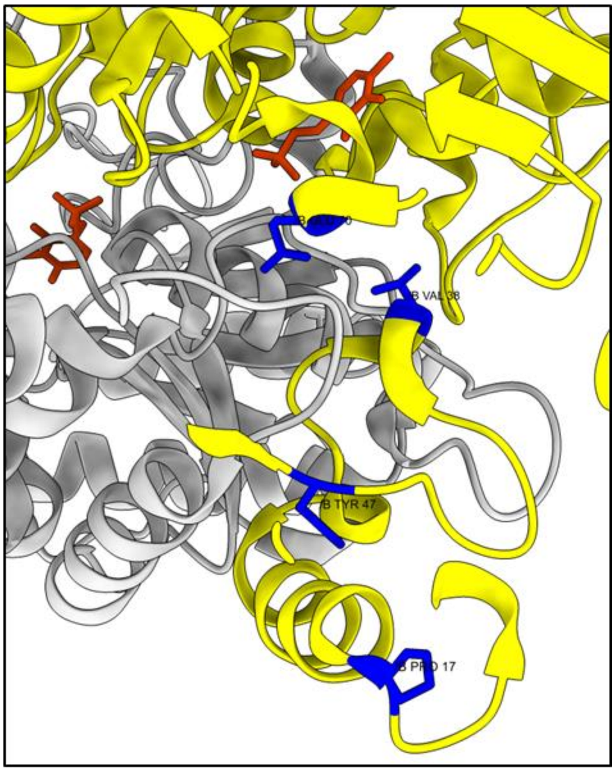

S17P T38V N70E

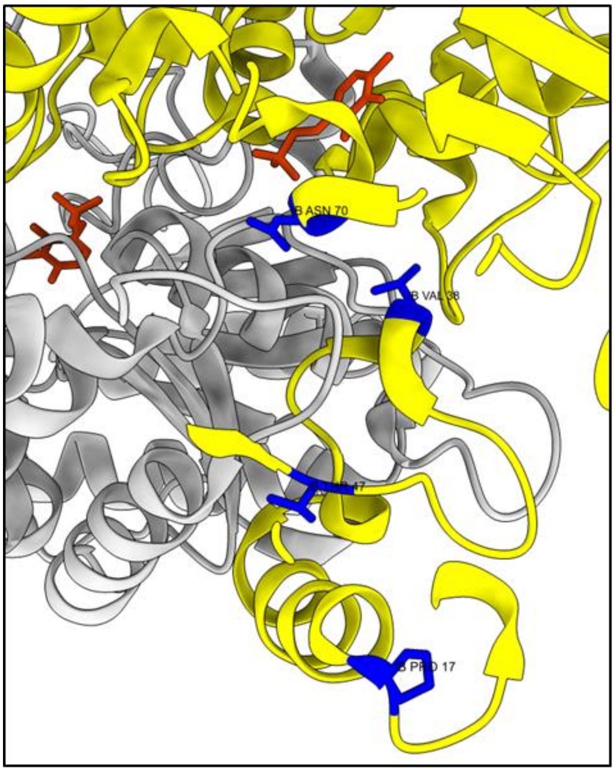

S17P T38V Y47T

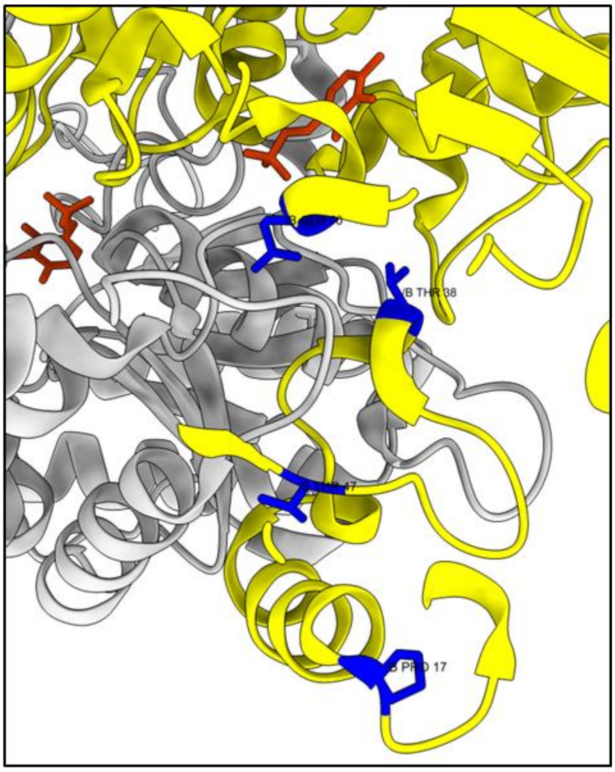

S17P Y47T N70E

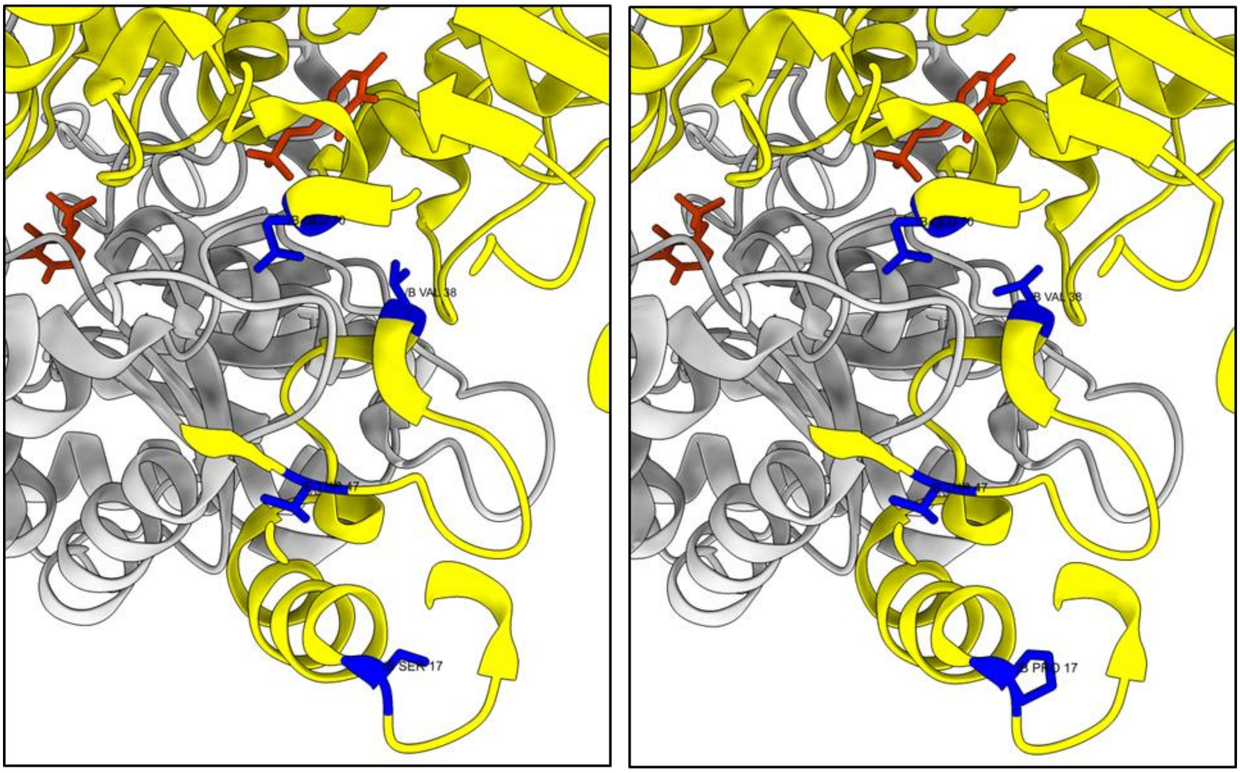

S17P T38V Y47T — S17P T38V Y47T N70E

Table S1: Used primers for the adaption to the motif.

Modification	Primer forward	Primer reverse
S17P	gtctatcgcgaaccagggccggccgccgccgatctg	cagatcggcggcggcaggccctggttcgcgatagac
T38V	gggaacacccgtaccgtggtttattcggcaccgtat	atacggtgccgaataaacccggtacgggtgttccc
N70E	gatttcgttaatgaatacaccgccctgattcatggg	cccatgaatcagggcggtgtattcattaacgaaatc
Y47T	tcggcaccgtatccccctaccgcagctcgcggccgg	ccggccgcgagctgcggtagggggatacggtgccga

*Table S2*Annealing temperatures for all primer pairs.

primer pair	Tm [°C]
1 and 3	58
2 and 6	62
1 and 5	58
4 and 6	62
1 and 8	58
7 and 6	62
1 and 10	58
9 and 6	62
1 and 12	58
11 and 6	62
7 and 10	72
7 and 12	72

9 and 12	72

Table S3: Used buffers for the determination of pH optima. All buffers had a concentration of 1 M.

PH	Buffer
2	Glycine hydrochloride
3	Citric acid potassium hydroxide
4	Citric acid potassium hydroxide
5	Potassium acetate
6	Potassium acetate
7	Potassium phosphate
8	Potassium phosphate
9	Potassium borate
10	Glycine potassium hydroxide
